# Supplementary material for: FADS1 and FADS2 Gene Polymorphisms Modulate the Relationship of Omega-3 and Omega-6 Fatty Acid Plasma Concentrations in Gestational Weight Gain: A NISAMI Cohort Study
Source: Nutrients. 2022 Mar 2;14(5):1056. doi: 10.3390/nu14051056 (PMC8912382; doi:10.3390/nu14051056)
Supplement: Supplementary file 1 [file nutrients-14-01056-s001.zip › nutrients-1549456-Supplementary Materials.pdf]

nutrients MDPI

Supplementary Materials:

Table S1. Characteristics of the FADS1 and FADS2 gene polymorphisms that will be used in the study on pregnant women from the NISAMI Cohort.

SNP	Gene	Chromosome Position[1]	Alleles[2]
(M/m)2			
rs174575	FADS2 intron1	61834531	C / G
rs174561	FADS1 intron1	61339284	T / C
rs3834458	Intergenic (FADS2)	61351497	T / Del[3]

[1]Chromosome position was obtained from the National Center for Biotechnology (NCBI, 2015).[2] M = major allele; m = minor allele. [3] Del = Deletion.

Table S2. FADS1 and FADS2 gene polymorphisms by anthropometric profile in the study on pregnant women from the NISAMI Cohort. Santo Antonio de Jesus, Bahia. 2013–2015

No excess weight gain	Excess gestational weight gain	p-value *	No excess weight gain	Excess gestational weight gain	p-value *
**Gene**	**N**	**%**	**N**	**%**	**p value ***
**SNP FADS2 rs174575**					
MM	50	48 .15	33	52 .27	0 .279
Mm e mm	66	51 .85	36	47 .73	
**SNP FADS 1 rs174561**					
MM	70	61 .11	42	62 .50	0 .903
Mm e mm	46	38 .89	27	37 .50	
**SNP FADS 2 rs3834458**					
MM	67	59 .26	39	57 .95	0 .776
Mm e mm	49	40 .74	30	42 .05	

n= 85:555 observations. Abbreviations: FADS: Desaturase gene polymorphisms; Del, deletion; HWE, Hardy-Weinberg equilibrium; MAF, Minor allele frequency; MM, high-frequency homozygote; mm, low-frequency homozygote;Mn, heterozygote; SNP, single nucleotide polymorphism.*Derived from the chi-square test.

Table S3. Characteristics of polymorphisms of the desaturase gene (FADS) in the sample of pregnant women followed by the NISAMI Cohort. Santo Antonio de Jesus, Bahia. 2013–2015.

Gene	Genotype (%)	MAF (%)
	MM	Mm
**FADS2**		
rs174575	CC (44 .9)	CG (45 .9)
**FADS 1**		
rs174561	TT (60 .5)	TC (33 .5)
**FADS 2**		
rs3834458	TT (57 .3)	TDel (34,6)

[1] n = 185: 555 observations. Abbreviations: FADS: Desaturase gene polymorphisms; Del, deletion; HWE, Hardy-Weinberg equilibrium; MAF, Minor allele frequency; MM, high-frequency homozygote; mm, low-frequency homozygote;Mn, heterozygote; SNP, single nucleotide polymorphism.a Derived from the chi-square test of the Hardy-Weinberg equilibrium.
